# Effect of bovine milk fat globule membranes as a complementary food on the serum metabolome and immune markers of 6-11-month-old Peruvian infants

**DOI:** 10.1038/s41538-018-0014-8

**Published:** 2018-04-12

**Authors:** Hanna Lee, Nelly Zavaleta, Shin-Yu Chen, Bo Lönnerdal, Carolyn Slupsky

**Affiliations:** 1 Department of Food Science and Technology, University of California, Davis, Davis, CA USA; 2 Instituto de Investigación Nutricional, Lima, 18-0191 Peru; 3 Department of Nutrition, University of California, Davis, Davis, CA USA

**Keywords:** Metabolomics, Developing world

## Abstract

This study builds on a previous study by this group in which 6–11-month-old Peruvian infants who were fed bovine milk fat globule membrane (MFGM) containing complementary food had significantly fewer episodes of infection-related bloody diarrhea relative to those consuming a control food (skim milk powder). Micronutrient deficiencies including zinc deficiency were prevalent in this study population. To understand the mechanism behind the health benefits of consuming MFGM, the serum metabolome and cytokine levels, as markers for systemic immune responses, were evaluated using ^1^H nuclear magnetic resonance-based metabolomics and a multiplex system, respectively. Combined with data on micronutrient status and anthropometry, a comparative analysis was performed. Supplementation with MFGM tended to improve micronutrient status, energy metabolism, and growth reflected as increased levels of circulating amino acids and weight gain, particularly in female infants compared to controls. Decreased levels of the microbial choline metabolite trimethylamine-*N*-oxide in the MFGM-supplemented group (both male and female infants) suggest a functional perturbation in the intestinal microbiota. A cytokine shift toward a less T helper type 1 response was observed in those receiving the MFGM supplement, which was mainly attributed to decreases in interleukin-2 levels. Our findings suggest that consumption of MFGM with complementary food may reverse the metabolic abnormalities found in marginally nourished infants, thereby improving metabolic regulation, which may lead to enhanced immunity.

## Infant nutrition: milk fat globule membrane reverses metabolic abnormalities

Bovine milk fat globule membrane (MFGM)-enriched complementary food has been recognized to be capable of reducing the episodes of bloody diarrhea in marginally nourished infants. However, the metabolic influence of MFGM remains unknown. Carolyn Slupsky from University of California-Davis and co-workers studied the impacts of bovine MFGM consumption on the levels of serum metabolome and immune markers. They found the supplements of MFGM improved the micronutrient status, energy metabolism, and growth, especially prominent for female infants. Moreover, MFGM complementary food lowered the level of the bacterial choline metabolite trimethylamine-N-oxide in the infants, indicating a functional perturbation to their intestinal microbiota. These findings suggest MFGM in complementary food can help to regulate the metabolic abnormalities of the infants with micronutrient deficiency, probably resulting in enhanced immunity.

## Introduction

Despite improving trends, diarrhea remains a global burden and one of the leading causes of death among children under the age of 5 years, accounting for 21% of total deaths worldwide.^1^ Less developed countries often suffer from high mortality rates mainly due to insufficient potable water facilities,^2^ poor sanitation, and micronutrient deficiencies (e.g., zinc) that render these populations at increased risk.

During the period when infants are exposed to complementary foods, the immune-protective properties of breast milk fade, even though the immune function of the infant has not yet fully developed. At this stage, a large load of antigens, as a result of exposure to new foods and bacteria, begin to be introduced into the digestive tract, making infants vulnerable to infection.^3^ Pathogenesis of gastrointestinal (GI) infection and related diseases (i.e., diarrhea) can begin with a dysregulated intestinal barrier and/or dysbiosis of the gut microbiota. Continuous failure of resistance to infection can lead to developmental challenges in infants. Thus, proper feeding and nutritional strategies during this period play key roles in shaping a healthy GI tract that ensures proper digestive and absorptive functions as well as a strong intestinal barrier and immune function.^4^

Maturation of the GI tracts occurs with the establishment of gut microbiota, which is a stepwise process that is orchestrated over time by interactions with environmental (or dietary) cues, host intestinal cells, as well as indigenous and foreign microorganisms. In this context, milk fat globule membrane (MFGM) has received considerable attention as a potential key nutrient to help modulate the gut microbiota and GI environment, but it has been historically removed during production of milk protein sources used in infant formulas.^5^

MFGM embraces hundreds of membrane-specific proteins and polar lipids that are derived from the mammary gland epithelium. Along with other milk nutrients, MFGM contains several elements (e.g., lactadherin,^6^ gangliosides, sphingolipids,^7^ and transforming growth factor-alpha^8^) that support growth and structural development of the intestine. In particular, sphingomyelin has been shown to increase the area of myenteric plexus, a part of the enteric nervous system that is responsible for GI tract motility.^9^ The protective activities of MFGM against pathogen and viral infections have also been proposed that include acting as a glycan decoy (e.g., MUC1, PAS 6/7, lactadherin, and glycolipids), or a bactericidal agent (e.g., xanthine oxidase).^10–12^ Milk gangliosides are predominantly associated with the MFGM fraction, the glycan moieties of which can interact with pathogens.^13^ Milk fat globule–epidermal growth factor 8 (lactadherin) contains cell-binding motifs (e.g., Arg-Gly-Asp sequence and Discoidin/F5/8C domains) that modulate cellular events including interference with rotavirus binding,^14^ intestinal mucosal repair, and phagocytic clearance of apoptotic cells.^15^ Interestingly, accumulation of host defense proteins on the MFGM surface was reported during *Staphylococcus aureus* infection of the mammary gland, which is reflective of a mammary gland immune response.^16^

In this study, Peruvian infants received either MFGM-enriched protein fractions or skim milk powder, both supplemented with one recommended dietary allowance (RDA) of micronutrients, twice daily for 6 months starting at approximately 6 months of age in a double-blind randomized controlled trial. We previously reported that infants consuming the MFGM-enriched supplement, as compared with those receiving a skim milk powder supplement, had significantly reduced episodes of bloody diarrhea.^17^ Given that the metabolic impacts of MFGM consumption in this cohort have not been explored, we conducted a metabolome screen of sugars, organic acids, amino acids, vitamins, alcohols, and other metabolites as well as the levels of 10 cytokines in these children before and after supplementation to determine if MFGM impacts overall metabolism and immunity. Further, data on micronutrient status (e.g., hemoglobin, folate, zinc, ferritin, and vitamin B12) and anthropometry (e.g., weight and length) collected from the trial were integrated for sub-group analyses. We hypothesized that consumption of MFGM-enriched complementary food alters metabolic pathways to enhance immunity and reduce infection-related diarrhea.

## Results

### Impact of MFGM-enriched protein supplementation on the serum metabolome

**Fig. 1:**
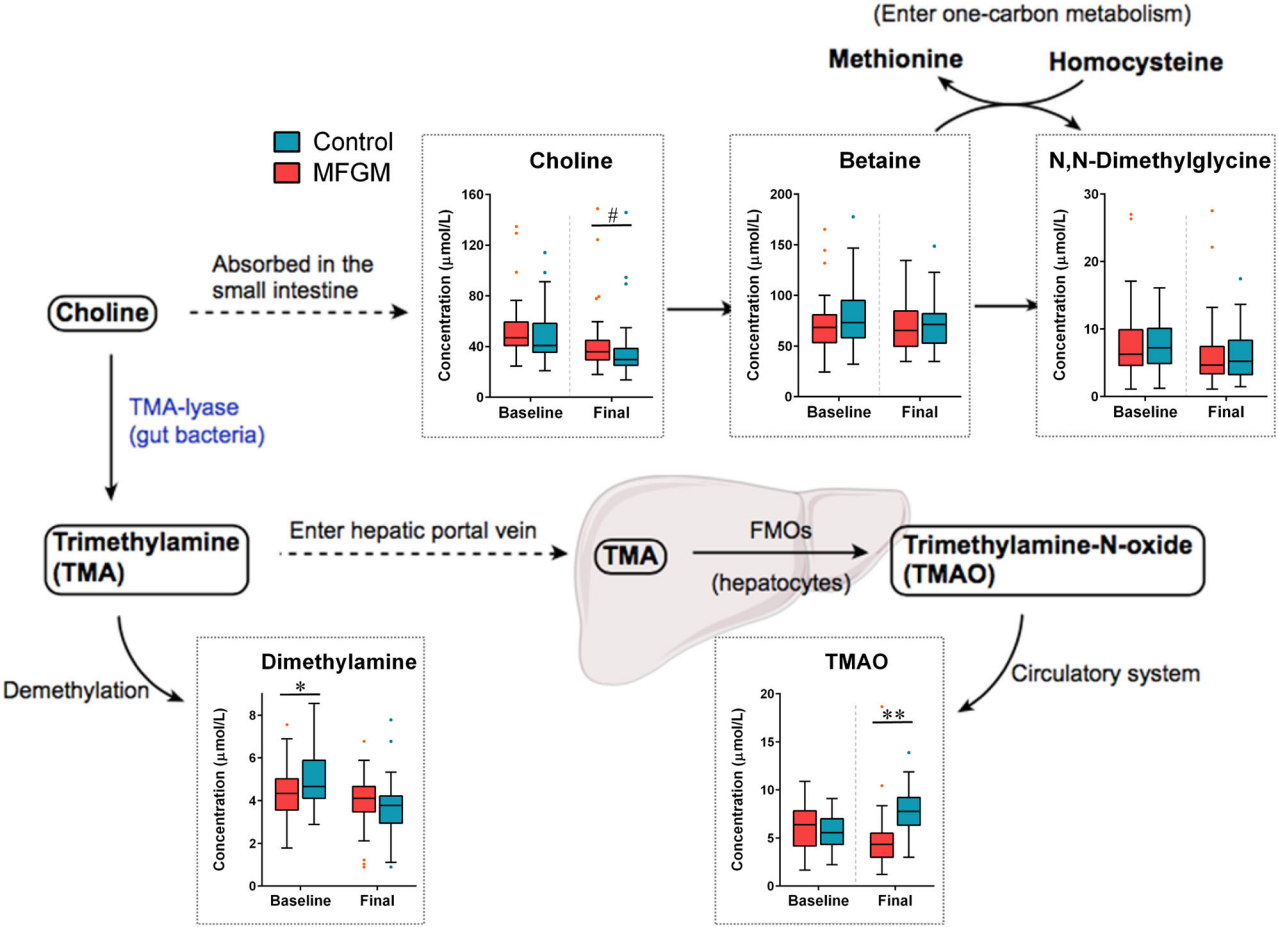
MFGM supplementation with complementary food for 6 months significantly alters host-microbial choline metabolism. Data are presented as median (solid line) ± interquartile range (IQR) in the box plot, and whiskers extend to the 1.5 IQR values. Group differences with approaching significance (0.05 < ^#^*P* < 0.1) or significance (0.1 < *P < 0.05, **P < 0.01) are noted. *TMA* trimethylamine, *TMAO* trimethylamine-N-oxide, *FMO* flavin-containing monooxygenase

Comparison of the serum metabolome between infants consuming MFGM and those receiving control (skim milk powder) supplement revealed that several metabolites were affected by MFGM supplementation at the end of the 6-month supplementation period that included leucine, lysine, valine, alanine, arginine, proline, tyrosine, ornithine, urea, aspartate, choline, mannose, pyroglutamate, and taurine, which increased in MFGM-supplemented infants, as well as acetone, 3-OHB (3-hydroxybutyrate), phenylalanine, and trimethylamine-*N*-oxide (TMAO), which decreased in MFGM-supplemented infants with significance (unadjusted *P* < 0.05) or approaching significance (unadjusted 0.05 < *P* < 0.10) (Fig. 1 and Table 1). Significance of diet effect was adjusted for sex (see Methods).

**Table 1:** Concentration differences of the serum metabolites between infants receiving either skim milk powder (control) or the MFGM supplement (MFGM) for 6 months. Only those metabolites with significance (unadjusted *P* < 0.05) or approaching significance (unadjusted 0.05 < *P* < 0.10), and moderate to strong effect sizes (Cliff’s Delta ≥ 0.28) are reported

Metabolite	All infants			Male Infants			Female Infants		
	^†^*P*-value	Effect size	MFGM > Control	*P*-value	Effect size	MFGM > Control	*P*-value	Effect size	MFGM > Control
Essential amino acids									
Isoleucine	ns	low	>	ns	low	>	0.085^#^	0.335	>
Leucine	0.039	low	>	ns	low	>	0.045*	0.389	>
Lysine	0.045	low	>	ns	low	>	0.009**	0.500	>
Phenylalanine	0.069	low	<	ns	low	<	ns	low	>
Threonine	ns	low	>	ns	low	<	0.080^#^	0.341	>
Valine	0.065	low	>	ns	low	>	0.024*	0.435	>
Non-essential amino acids									
Alanine	0.039	low	>	ns	low	<	0.056^#^	0.371	>
Arginine	0.049	low	>	ns	low	>	0.049*	0.382	>
Asparagine	ns	low	<	0.094	0.275	<	ns	low	<
Proline	0.038	low	>	ns	low	<	0.040*	0.400	>
Serine	ns	low	>	ns	low	<	ns	low	>
Tyrosine	0.080	low	>	ns	low	>	0.052^#^	0.377	>
Urea cycle intermediates									
Ornithine	0.034	low	>	ns	low	<	0.005**	0.529	>
Urea	0.075	0.383	>	0.021*	0.377	>	0.045*	0.388	>
Ketone body metabolites									
Acetone	0.020	low	<	ns	0.051	<	0.065^#^	0.359	<
3-OHB	0.026	low	<	ns	low	<	0.049*	0.382	<
Other metabolites									
Aspartate	0.078	low	>	ns	low	<	ns	0.288	>
Creatine	ns	low	>	0.055^#^	0.315	>	ns	low	>
Choline	0.090	0.307	>	ns	low	>	0.048*	0.385	>
DMA	ns	low	>	0.042*	0.333	>	ns	low	>
Mannose	0.091	0.329	>	0.029*	0.360	>	ns	low	>
*N*,*N*-DMG	ns	low	<	ns	low	<	ns	low	>
PG	ns	low	<	ns	low	<	0.046*	0.388	<
Pyroglutamate	0.077	low	>	ns	low	>	0.026*	0.429	>
Succinate	ns	low	>	0.067^#^	0.301	>	ns	low	>
Taurine	0.083	low	>	ns	low	>	0.080^#^	0.341	>
TMAO	<0.001**	0.749	<	<0.001**	0.696	<	<0.001**	0.841	<

^†^*P*-values were sex adjusted (see Methods)

*3-OHB* 3-hydroxybutyrate, *DMA* dimethylamine, *N,N-DMG* *N*,*N*-dimethylglycine, *PG* propylene glycol, *TMAO* trimethylamine-*N*-oxide Group differences with approaching significance (0.05 < #*P* < 0.1) or significance (0.1 < *P < 0.05, **P < 0.01)

The effect of the MFGM supplement appeared to be stronger in female infants for some metabolites that included isoleucine, leucine, lysine, threonine, valine, alanine, arginine, proline, tyrosine, ornithine, urea, choline, pyroglutamate, and taurine, which were higher, and 3-OHB, acetone, and propylene glycol, which were lower, in female infants with MFGM supplementation with moderate (0.28–0.43) to strong (>0.43) effect sizes (Table 1). In male infants consuming the MFGM supplement, metabolites with significance or approaching significance with a moderate effect size were creatine, dimethylamine, mannose, and succinate, all of which increased. While these metabolite concentrations appeared to be significant in one sex, similar trends were observed in the other sex with MFGM supplementation.

These metabolites could be classified into several pathways including ketone body metabolism, which tended to decrease in the MFGM-supplemented group, the urea cycle, which tended to increase in the MFGM-supplemented group, and amino acid metabolism, which also tended to increase in the MFGM-supplemented group.

Interestingly, the control group had significantly higher levels of the bacterial choline metabolite TMAO relative to the MFGM group post intervention with a large effect size (unadjusted *P* < 0.001, effect size = 0.75; Fig. 1). Choline is absorbed in the small intestine, and later converted to betaine to enter one-carbon metabolism, or be anaerobically metabolized by gut bacteria to trimethylamine, which is transported through enterohepatic flow and further oxidized to TMAO by the liver enzyme FMO (flavin-containing monooxygenase). Choline and dimethylamine levels in serum were lower in those infants (control group) post intervention (not significant, unadjusted *P* > 0.05; effect size = 0.31 and <0.28, respectively). Quantified metabolite concentrations (median and interquartile range) are summarized in Supplementary Table S3.

### Impact of MFGM-enriched protein supplementation on serum cytokine profiles

**Fig. 2:**
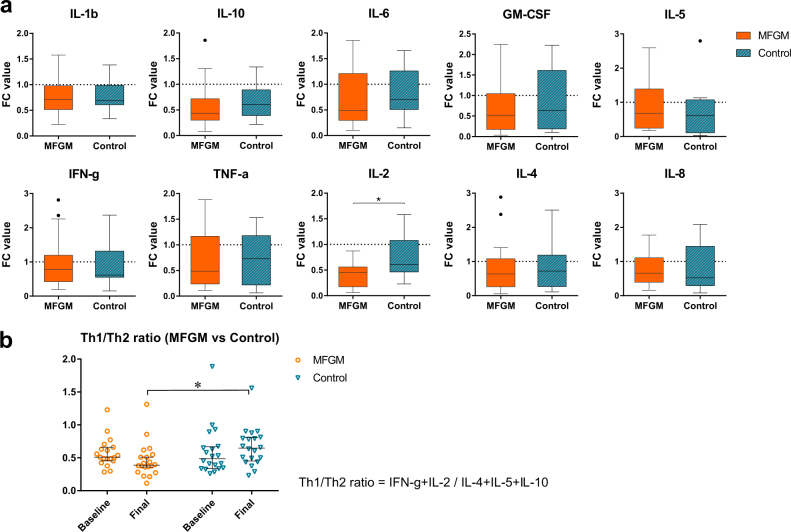
Serum cytokine levels of infants in the MFGM (orange) and the control (blue) groups. **a** Fold change of each cytokine from baseline assessment was calculated as FC (post-/pre) value (expressed as median ± interquartile range). IL-2 concentrations were significantly decreased in the MFGM supplemented group. **b** Th1/Th2 ratio (defined as the ratio of Th1 (IFN-γ, and IL-2) to Th2 cytokines (IL-4, IL-5, and IL-10)) was significantly different between groups after supplementation (*P < 0.05; Mann-Whitney) (expressed as median with 95% Cl)

The levels of 10 cytokines measured in serum all showed decreasing trends starting from baseline toward the end of the study regardless of which group they belonged (Fig. 2). The cytokine levels at both time points were not significantly different between groups because of large variations in cytokine levels of individuals within each group (coefficient of variation varying from 26.5 to 162.1%). It is important to note that blood samples were drawn only when infants were healthy, and not showing any pathological symptoms (e.g., diarrhea), which indicates an absence of controlled (or artificial) immune stimulus. Biancotto et al.^18^ reported that due to the inter-individual variations in basal cytokine levels, a baseline measurement of each individual can serve as a better control than comparing two different cohorts at a single time point. In this context, fold change (FC) values of cytokines were calculated and compared between groups (where a FC of one means there is no change between baseline and final). As shown in Fig. 2a, the interleukin (IL)-2 levels decreased more in the MFGM-supplemented group (median FC value: 0.457) compared with the control group (median value: 0.609) (unadjusted *P* = 0.004, Benjamini–Hochberg false discovery rate (B-H FDR) adjusted *P* = 0.039; Mann–Whitney *U*-test). While not significant (unadjusted *P* = 0.108, B-H FDR adjusted *P* = 0.540), decreases in IL-10 levels were greater in the MFGM (median value: 0.433) relative to the control group (median value: 0.608).

To investigate the patterns in cytokine profiles, the dominance of T-helper 1 (Th1; cell-mediated) activity or T-helper 2 (Th2; humoral mediated) activity was estimated by the Th1/Th2 ratio defined as the ratio of Th1 (e.g., interferon-γ (IFN-γ) and IL-2) to Th2 cytokines (e.g., IL-4, IL-5, and IL-10).^19^ As shown in Fig. 2b, the Th1/Th2 ratio of the MFGM group (orange circles) decreased from 0.58 ± 0.05 to 0.46 ± 0.08, showing a possible shift toward Th2 dominance. In contrast, the ratio of the control group (blue triangle) increased from 0.59 ± 0.06 to 0.66 ± 0.06, which was significantly different from the MFGM group at the final time point (unadjusted *P* = 0.007, B-H FDR adjusted *P* = 0.015; Mann–Whitney *U-*test).

### Association between the serum metabolome, cytokines, and micronutrient status

**Fig. 3:**
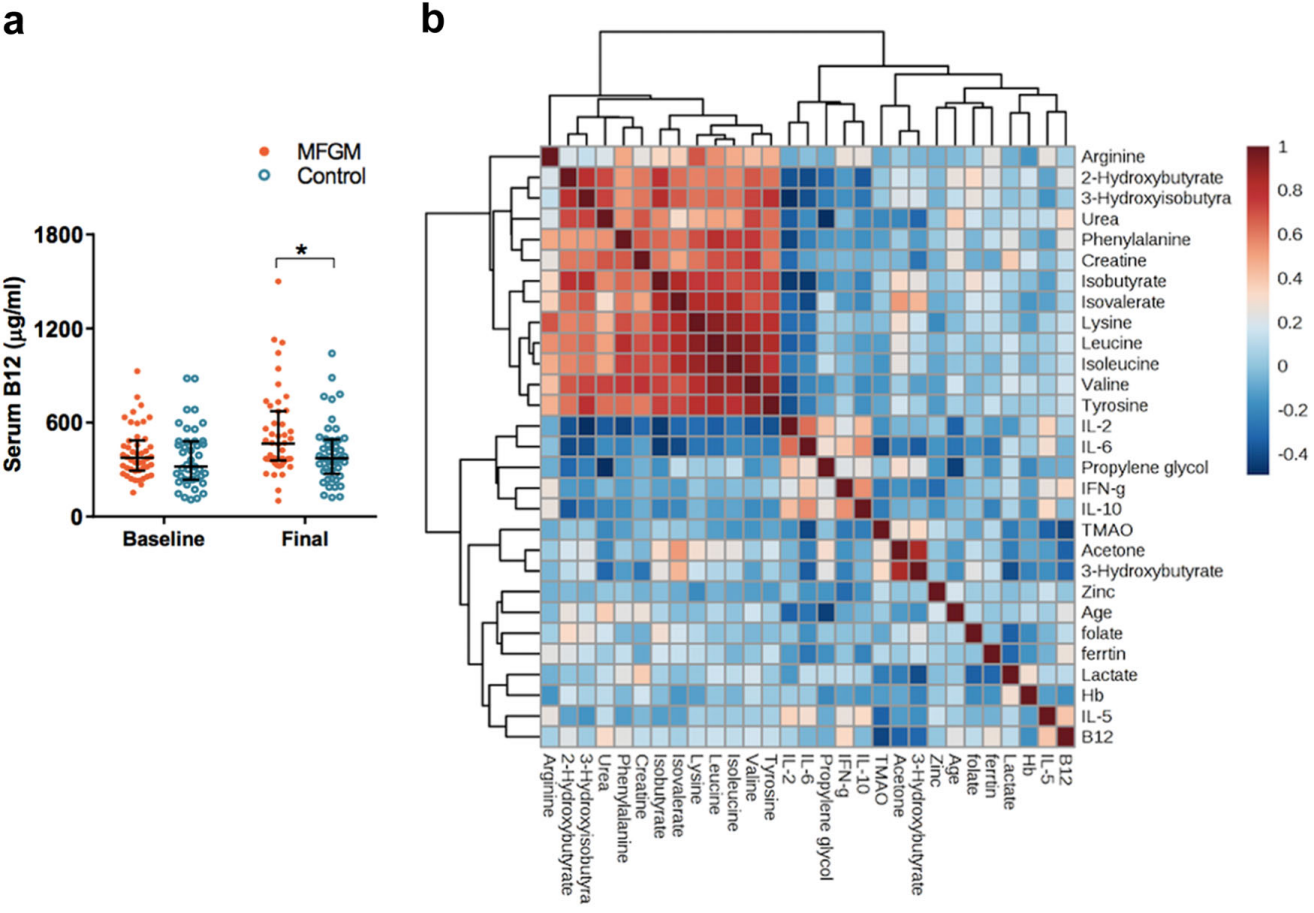
**a** Serum vitamin B12 was significantly higher in the MFGM group (orange filled circle) compared with the control group (blue empty circle) after 6-months of micronutrient supplementation. Data are presented as median (solid line) ± interquartile range (IQR). **b** Spearman rank correlation between the serum metabolites, cytokines, and micronutrient concentration post-intervention. Data showing at least moderate correlation (|*ρ*| > 0.4) were selected. The intensity of color represents the magnitude of the correlation, and the color indicates either positive (red color; 0 < *ρ* < 1), or negative correlations (blue color; −0.5 < *ρ* < 0). *ρ* = correlation coefficient

Analysis of micronutrient status at baseline and after supplementation revealed that serum vitamin B12 levels were significantly increased in the MFGM-supplemented group (unadjusted *P* = 0.020, Mann–Whitney *U*-test; effect size = 0.269), even though both groups were given the same one RDA of a micronutrient mixture during the intervention (Fig. 3a). To determine if significant correlations exist between the serum metabolome, cytokine profiles, and micronutrient status (including serum hemoglobin, folate, zinc, ferritin, and vitamin B12), Spearman's rank correlation analysis was performed (Fig. 3b). *P-*values and coefficients (*ρ*) are summarized in Supplementary Table S5. No metabolites correlated with TMAO (*ρ* < 0.30), but vitamin B12 and IL-6 levels were negatively correlated (*ρ* = −0.42 and −0.41, respectively). Interestingly, IL-2 was inversely correlated with the concentrations of several amino acids and related metabolites that included urea, creatine, valine, tyrosine, isoleucine, leucine, lysine, and phenylalanine at moderate levels (−0.50 < *ρ* < −0.30). Also, a strong positive correlation was observed between IL-2 and IL-6 (*ρ* = 0.63). As expected, several amino acids (lysine, leucine, isoleucine, phenylalanine, tyrosine, and valine), and amino acid breakdown products (isobutyrate, isovalerate, 2-hydroxybutyrate, and 3-hydroxyisobutyrate) were highly correlated with each other (*ρ* > 0.60), including all three branched chain amino acids (BCAAs), which had strong, positive correlations (*ρ* > 0.90). The two ketone metabolites, acetone and 3-hydroxybutyrate, were also strongly positively correlated (*ρ* = 0.85) with each other, and negatively correlated with vitamin B12 (*ρ* = −0.33 and −0.31, respectively). Positive correlations between vitamin B12 and IL-5 (*ρ* = 0.40) or IFN-γ (*ρ* = 0.34) were also observed. Serum zinc had a weak negative correlation with IFN-γ (*ρ* = −0.30). It is important to note that correlation does not imply causation. Nonetheless, these correlations help explain our observations as discussed below.

### Sex differences

**Fig. 4:**
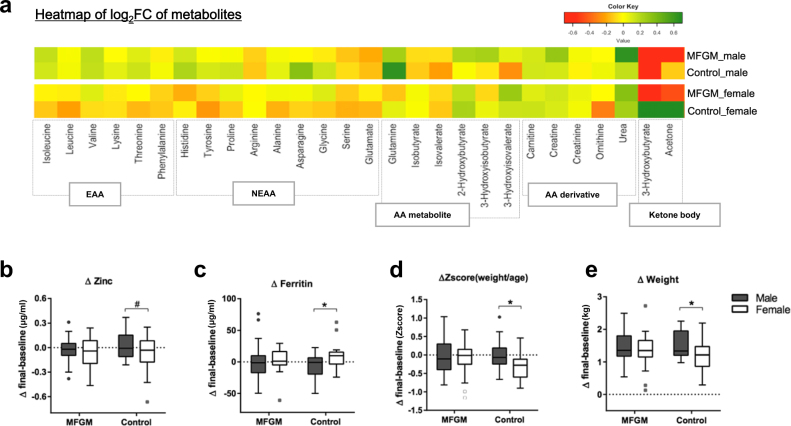
Male (*n* = 28 (MFGM); 24 (control)) and female infants (*n* = 18 (MFGM); 21 (control)) showed different responses to MFGM supplementation. **a** Heatmap showing the log_2_FC of serum metabolites associated with amino acid metabolism. Color code indicates negative (red) to positive (green) changes. Changes in the serum levels of **b** Zinc (μg/ml), and **c** ferritin (μg/ml); and changes in (**d–e**) anthropometric data (weight expressed as kg) calculated by subtracting the initial values from the final values of measurements are shown with identified sex differences (0.05 < ^#^*P* < 0.1; *P < 0.05; **P < 0.01). Data are presented as median (solid line) ± interquartile range (IQR) in the box plot, and whiskers extends to the 1.5 IQR values. *EAA* essential amino acid, *NEAA* non-essential amino acid, *AA* amino acid

As mentioned above, female infants receiving the control supplement appeared to have a trend toward depletion of essential and non-essential amino acids, whereas this trend was not observed in male infants as shown in the heatmap of metabolite fold changes (log_2_ FC) from baseline to 6-month post intervention (Fig. 4a). Serum cytokine levels were not impacted by sex. Although MFGM supplementation had no impact on zinc levels, there were differences in zinc levels based on sex. The baseline level of serum zinc was higher in female infants (*P* = 0.005, Mann–Whitney *U-*test; effect size = 0.277; data not shown), which decreased to the same level as male infants at the final time point. A significant Time×Sex interaction for serum zinc was observed (*P* = 0.037, 2-way repeated measures analysis of variance (ANOVA) on log-transformed data; data not shown). Comparison of the change in serum zinc from baseline to final revealed that female infants in general tended to have decreased serum zinc relative to males within the control group (*P* = 0.087, Mann–Whitney *U-*test; effect size = 0.226; Fig. 4b) but not within the MFGM group (*P* > 0.05; Mann–Whitney *U*-test). There were no differences between groups with respect to serum ferritin at each time point (data not shown), but the change in ferritin was higher in female infants compared with males, where the difference was only significant within the control group (male < female, *P* = 0.018; effect size = 0.435; Fig. 4c). The observed Spearman's correlation patterns in Fig. 3b did not show sex differences, although the magnitude of correlation coefficients (*ρ*) differed based on sex.

In terms of anthropometric measures, significant sex differences were observed for body weight but not for body length. Figure 4d shows that female infants had lower Δ*Z-*score (weight-for-age) values than males, which was significant within the control (male > female, *P* = 0.014; effect size = 0.425) but not within the MFGM group (male = female, *P* = 0.661). Consistent findings were observed with the calculated Δ weight values (male > female, *P* = 0.022; effect size = 0.341 within the control, *P* = 0.423 within the MFGM; Fig. 4e) despite the comparable Δ length values between sexes (data not shown). These results agreed with the serum amino acid profiles, which were found to be lower in the female infants of the control group.

## Discussion

In this study, the most notable alterations in the post-intervention serum metabolome induced by the MFGM supplementation were increased urea cycle metabolites, as well as decreased ketone bodies and TMAO relative to infants receiving control supplement. This study was conducted in a rural area of Peru, where infants had inadequate nutrition, suffering from stunting, multiple micronutrient deficiencies, and infectious diseases. Significant sex differences in growth parameters were observed (i.e., weight-for-age *Z*-score (WAZ) and/or final weight; males > females) when analyzed within the control group, which was not significant within the MFGM group. In the previous report, differences in growth between the MFGM and control groups were not reported as both sexes were included in the statistical analyses.^17^ Interestingly, there was a decrease in essential and non-essential serum amino acids (compared with baseline) in the female control group after the intervention, which was not evident in the male group. A correlation between WAZ and decreased serum amino acids was previously observed in Malawian children who were stunted (height­-for-age *Z*-score < −2) with all nine essential amino acids significantly lowered in their serum.^20^ We did not find any significant differences in body length, but infants with severe stunting (*Z*-scores < −2) were initially excluded from baseline screening.

The basis for the observed sex differences in the amino acid pool at this age is not yet known. One potential reason may involve inducible nitric oxide synthase (iNOS) production. Zinc has been shown to be a potential regulator of iNOS activity in inflammation via inhibition of nuclear factor-κB activity.^21^ In our study, we observed significant decreases in serum zinc in control females at the final time point compared with males. Zinc supplementation has been shown to increase hepatic activity of ornithine transcarbamoylase, a key enzyme in the urea cycle.^22^ This could mean that more arginine is metabolized to creatine and urea in males, resulting in lower iNOS expression, whereas in females, this pathway is suppressed. Indeed, more than 80% of the infants in the trial were zinc deficient at enrollment (serum zinc < 10.7 μmol/L).^17^ Zinc deficiency impedes normal growth and tissue repair^23^ and is a major health concern, as it can lead to immune dysfunction and increased susceptibility to infectious disease. A burst of cellular demand for zinc during an infection depletes the body’s zinc stores and can accelerate disease pathology resulting in a vicious cycle.^24^ Interactions between body zinc and protein in less-nourished conditions are bidirectional and very complex.^25^ Zinc deficiency has been implicated in impaired protein and amino acid metabolism by inhibiting protein synthesis, although whether it decreases or increases the serum amino acid pool is not entirely clear.^26,27^ Reduced urinary BCAA catabolites in zinc-deficient mice were previously reported.^28^ Our data showed that serum zinc levels were also weakly negatively correlated with serum levels of IFN-γ, suggesting that lower zinc and amino acids may be associated with increased infection. In this cohort, MFGM was shown to be protective against infection, including severe forms of diarrhea, such as bloody diarrhea,^17^ that requires prompt medical care to prevent further damage to intestinal mucosa and nutrient malabsorption.^2^

An important vitamin for neurological growth and development is vitamin B12. In this study, infants were generally deficient in vitamin B12 at baseline, but for those supplemented with MFGM, increased serum levels of vitamin B12 were observed at the final time point. Vitamin B12 is required for 1-carbon metabolism, converting homocysteine to methionine via methionine synthase, as well as the conversion of methylmalonyl-CoA to succinyl-CoA, which is used in the tricarboxylic acid (TCA) cycle through the enzyme methylmalonyl-CoA mutase. The latter is an important step to extract energy from oxidation of odd-chain fatty acids, cholesterol, and amino acids, and its inhibition caused by insufficient vitamin B12 may result in impairment of acetyl-CoA incorporation into the TCA cycle, which is then diverted to form ketone bodies.^29,30^ Levels of ketone body metabolites, 3-hydroxybutyrate and acetone, were observed to be significantly higher in control females relative to their control male counterparts and to the females in the MFGM group. Considering the lower vitamin B12 levels in the control females, this may in part explain the elevated ketone bodies in this group,^31^ which appeared to be partially compensated for by other factors in males (Fig. 4). Indeed, vitamin B12 and acetone were shown to be negatively correlated.

As humans cannot synthesize vitamin B12, increased levels are either attributed to dietary supply (e.g., animal products) or the gut microbiota. As the gut microbiota matures, microbial genes encoding vitamin B12 synthesis increase with age.^32^ Damage to the gut lining may result in poor vitamin B12 absorption. In a recent report, MFGM supplementation of formula provided to neonatal rat pups improved maturation of intestinal morphology and gut microbiota compared with pups fed control formula.^33^ Despite the higher serum choline levels in the MFGM group, higher levels of TMAO in the control group indicate potential perturbations in microbial metabolic activities in the gut because TMAO formation is obligate gut microbiota dependent.^34^ Higher fasting plasma levels of TMAO have been strongly associated with the risk of cardiovascular disease since it inhibits reverse cholesterol transport.^34^ Similar results have been reported in a protein-malnourished weaned mouse model with greater amounts of TMAO and lower amounts of BCAA catabolites excreted into urine.^28^ It was hypothesized that the inflamed state after protein deficiency could alter the gut environment to change microbial compositions. It is also plausible that improved intestinal absorption of choline (in the MFGM group) may subsequently reduce the choline flow to the colon to be metabolized by bacteria.

The decreased Th1 response in the MFGM group was mainly attributed to the significant decrease in IL-2 concentration, whereas decreased Th2 response in the control group was mainly due to a reduction in IL-5. We observed a positive correlation between IL-5 and vitamin B12 status, which agrees with a previous finding where vitamin B12 deficiency caused a cytokine shift toward a Th2 response.^35^ Although this is an oversimplification of a complex issue, the Th1 dominance is generally more aggressive, and caused by overstimulation of immune cells (i.e., natural killer cells or T cells), whereas the Th2 dominance can be seen in allergy or low-level systemic inflammation. IL-2 or IL-2 receptor expression is a rough index of Th1 cell proliferation, proposed as an indicator of stress level or frequency of upper respiratory infection.^36^ Feeding formula containing docosahexaenoic acid and arachidonic acid has been reported to reduce IL-2 secretion suppressing Th1-type reactions.^37^ In addition, a cytokine shift to Th1-dominant patterns was observed in vitro in peripheral blood mononuclear cells isolated from Tanzanian children who were zinc deficient.^38^ Propagation of immune responses such as immune cell proliferation, cytokine and antibody production, and tissue repair all require constant utilization of essential amino acids, which paralleled with the observed negative correlations between IL-2 levels and several AAs (valine, isoleucine, leucine, lysine, phenylalanine, and tyrosine). However, how MFGM consumption would modulate balance in the immune system needs further investigation.

In conclusion, our data suggest that consumption of bovine MFGM-supplemented food may reverse abnormalities in protein and energy (ketone body) metabolism found in marginally nourished (deficient in micronutrients) Peruvian infants as observed in the altered serum metabolome. Improved micronutrient status (i.e., zinc and B12) and decreased TMAO levels in those consuming the MFGM-supplemented food suggest functional changes in the intestinal microbiota, which may lead to enhanced immunity. Female infants tended to show a reduced serum amino acid pool along with less weight gain relative to male infants, which was improved in female infants provided the MFGM supplementation. Note that the micronutrient deficiencies (e.g., zinc) in the study population were likely to have caused poor digestion/absorption of macronutrients (e.g., proteins) due to reduced digestive enzyme activities, impaired absorptive mucosa, or frequent diarrheal disease.^25^ Yet, it is not possible to determine with the current data whether the observed benefits (e.g., amino acid and micronutrient status) with MFGM consumption came from increased inputs (availability) or decreased outputs (depletion). To improve complementary feeding strategies for infants in a population-specific manner, particularly for those who have less access to adequate nutrition, understanding the complexity of interactions between dietary components, host cells, and gut microbiota is critical. Based on the current findings, there are two possible pathways through which MFGM functions: (1) providing bioactive compounds to support intestinal maturation that improves mucosal absorptive capacity and immune systems; and/or (2) modulating gut microbial structure and/or function to impact systemic immunological and metabolic pathways. To understand specific health targets of MFGM as a nutraceutical, mechanistic studies on the effects of MFGM consumption at the tissue and/or cellular levels needs to follow.

## Methods

### Study design

This study was a 6-month, double-blind randomized controlled trial of Peruvian infants, carried out from 2004 through 2005 at Villa El Salvador, Peru (*n* = 550 enrolled; 499 completed). Details of the study design and original outcome measures were previously reported.^17^ Inclusion criteria were healthy infants living in the study area, ages 6–11 months, born at term with a birth weight ≥ 2500 g, and primarily breast-fed. Exclusion criteria were chronic disease, serious illness or congenital malformations, severe malnutrition (weight/height ≤ 2 *z* scores), and proven or probable milk allergy. At enrollment, infants were randomly assigned to one of two groups to receive two daily servings of a powdered meal containing 15% of protein either as bovine MFGM-enriched whey protein concentrate (Lacprodan^®^ MFGM-10, Arla Foods Ingredients, Denmark) (MFGM group), or bovine skim milk protein (control group) that was to be mixed into a complementary food. Except for the protein source, the macronutrient composition of the food was identical between the two groups. The powder (40 g; 192 kcal per day) consisted of 20 g carbohydrate, 9.6 g fat, 6 g of protein and 240 mg calcium. The powdered meal was also supplemented with 1 recommended dietary allowance (RDA) of multiple micronutrients for both groups to improve micronutrient deficiencies that are prevalent in the study population. The amino acid compositions of protein fractions for each of the powders are depicted in Supplementary Table S1. The serum samples used for the current study were collected at entry (baseline) and at the end of the study, and kept frozen until analysis. Additional information concerning study design and procedures are reported elsewhere.^17^

### Ethical considerations

The protocol was approved by the ethics committees of IIN, and the University of California, Davis. The protocol was authorized by the Instituto Nacional de Salud from the Ministry of Health, Lima, Peru. This study was conducted in accordance with the Declaration of Helsinki and Good Clinical Practice guidelines. Parents of participant children signed informed consent at entry.

### Sample preparation for serum metabolome analysis

100 samples (50 MFGM group + 50 control group) collected at two time points (baseline and after supplementation; total 200 samples) were selected from the original study population for metabolomics analysis by stratified random sampling to match selected parameters including sex, age at entry, and presence of anemia at entry in both groups (Supplementary Table S2). Prior to analysis, samples were coded as either Treatment-A or Treatment-B to reduce the risk of bias, and were unblinded after analysis was complete. Sample preparation procedures for NMR analysis was similar to what was described previously.^39^ Briefly, frozen serum samples were removed from −80 °C storage and thawed on ice, after which the samples were filtered through a 3 kDa cutoff filter (Amicon) at 4 °C to separate small soluble metabolites from non-polar metabolites, lipids, and proteins. To 207 μL of serum, 23 μL of an internal standard containing 5 mM 3-(trimethylsilyl)-1-propanesulfonic acid–d6 (DSS-d6) and 0.2% NaN_3_ in 99.8% D_2_O was added. The pH of the sample was adjusted to 6.8 ± 0.1 by the addition of small amounts of hydrochloric acid and sodium hydroxide to minimize pH-based chemical shift in the NMR spectrum, and 180 μL was transferred to a 3 mm NMR tube. Samples were stored at 4 °C until NMR data acquisition.

### NMR data acquisition and metabolite quantification

NMR spectra were acquired at 25 °C using the Bruker noesypr1d experiment on a Bruker Avance 600 MHz NMR spectrometer. ^1^H NMR spectra were acquired with a spectral width of 12 ppm, a total acquisition time of 2.5 s, 8 dummy scans, 32 transients and water saturation during the prescan delay (2.5 s) and mixing time (100 ms) to minimize impact of water on the spectrum. NMR spectra were zero-filled to 128k data points, Fourier Transformed with a 0.5-Hz line broadening, manually phased and baseline corrected. Metabolites were identified and quantified using Chenomx NMRSuite v8.0 (Chenomx Inc., Canada) using a concentration of internal standard (DSS) as previously described.^40^ Samples with excessive dilution, or noisy spectra were excluded from analysis.

### Serum metabolomic data analysis

Univariate analysis of individual metabolite concentrations comparing between the two groups at two time points (i.e., baseline and end of the study) was done for all infants together as well as in each sex separately. Since a large portion of metabolites deviate from a normal distribution (>75%) based on a Shapiro-Wilk test (*P* < 0.05) and in some cases variance was unequal between groups, non-parametric statistical tests were chosen to deal with the heteroscedasticity. Significance was assumed at *P* < 0.05, unless stated otherwise. To account for sex differences found in metabolite patterns, a linear regression model of transformed metabolite data (log-transformed to approximate normality) as a function of diet and sex using the “stats” package in R^41^ was constructed to evaluate the sex adjusted effects of diet on each metabolite level. A Sex*Diet interaction term was included in the model and additional statistical results from the linear model are summarized in Supplementary Table S4. Within each sex (i.e., males or females), comparison of individual metabolite concentrations between the two groups at two time points (i.e., baseline and end of the study) was done using the Mann–Whitney *U* test on raw metabolite data using GraphPad Prism (Version 5.0, GraphPad Software, San Diego, CA). To investigate the magnitude of the response to diet, non-parametric effect sizes for individual non-transformed metabolites were calculated using Cliff’s Delta computation in the “effsize” package in R.^42^ Heat-map analysis was performed using R version 3.2.1 for visualization of different patterns in metabolite fold changes (binary transformed to normalize the distribution) between sexes.

To identify the biological pathways or functions that are significantly enriched in quantified metabolomics data, both metabolite set enrichment analysis and metabolite pathway enrichment analysis were performed using the MetaboAnalyst analysis tool. Concentration data were log-transformed, mean-centered, and divided by the standard deviation of each variable for data normalization before the data input, after which the compound names were all matched with the reference database (e.g., HMDB). The results are presented in Supplementary Figure S6-7.

### Measurement of serum cytokines

80 serum samples were randomly selected (20 samples × two groups × two time points) and thawed immediately prior to analysis, and ten cytokines (GM-CSF, IFN-γ, IL-1β, IL-2, IL-4, IL-5, IL-6, IL-8, IL-10, and TNF-α) were simultaneously measured using a Human Cytokine Ultrasensitive Panel for Luminex™ Platform (Invitrogen, CA) according to the manufacturer’s instructions. Samples were briefly centrifuged to remove any aggregates, and were frequently vortexed during the assay. All samples were analyzed in duplicate, and the final values were calculated as medians of 100 beads for each cytokine that were read on the Luminex 100TM platform (Luminex Co., TX). The sensitivity of each cytokine assay was less than 1 pg/mL. Fold changes of cytokine levels (post/pre) were calculated and subjected to univariate analysis (Mann–Whitney *U* test) to test effect of diet. The ratio of Th1 (e.g., IFN-γ and IL-2) to Th2 cytokines (e.g., IL-4, IL-5, and IL-10) was determined by calculating the sum of Th1 cytokines and dividing by the sum of Th2 cytokines,^19^ followed by Mann–Whitney *U* tests for diet comparison.

### Data integration and correlational analysis

To extend our understanding of the current findings, clinical data (e.g., micronutrient status and anthropometric measurement) collected during the trial^17^ were integrated into the current analysis. Data were re-calculated for the selected infants used in the current study and statistical differences based on diet and sex were analyzed. Univariate analyses of clinical data were performed (Mann Whitney-U on original data, and Time*Sex 2-way ANOVA on log-transformed data to approximate normality prior to analysis), and identified significance was reported. Correlations between the serum metabolome, cytokines, and micronutrient status were analyzed by Spearman’s rank test to investigate the statistical dependencies between the rankings of two sets of variables using GraphPad Prism and MetaboAnalyst 3.0 (www.metaboanalyst.ca/).

### Data availability

Data generated and/or analyzed to support the findings of this study are included in this published article and its supplementary files. Other relevant data including raw metabolomics data (NMR spectra), cytokine data, and human subjects data are available from the corresponding author on request.

## Electronic supplementary material

Supplementary Data(PDF 954 kb)

## Electronic supplementary material

**Supplementary information** accompanies the paper on the *npj Science of Food* website (10.1038/s41538-018-0014-8).
