# Supplementary material for: Effect of bovine milk fat globule membranes as a complementary food on the serum metabolome and immune markers of 6-11-month-old Peruvian infants
Source: NPJ Sci Food. 2018 Apr 12;2:6. doi: 10.1038/s41538-018-0014-8 (PMC6550191; doi:10.1038/s41538-018-0014-8)
Supplement: Supplementary file 1 — Supplementary Data(PDF 954 kb) [file 41538_2018_14_MOESM1_ESM.pdf]

Supplementary Figures/Tables for

Effect of bovine milk fat globule membranes as a complementary food on the serum metabolome and immune markers of 6-11-month-old Peruvian infants

Hanna Lee[1], Nelly Zavaleta[2], Shin-Yu Chen, Bo Lönnerdal[3], Carolyn Slupsky*[1, 3]

[1]*Department of Food Science and Technology, University of California-Davis, CA, USA*

[2]*Instituto de Investigación Nutricional, Lima, Peru 18-0191*

[3]*Department of Nutrition, University of California-Davis, CA, USA*

Supplementary Table S1. Amino acid composition of the protein fraction of each powder meal. Complementary powder meals were given twice daily (40 g/d) in addition to the regul ar meals of infants.

Amino acid	unit	Calculated on estimated g /100 g powder	
		Control food	MFGM-enriched food
Isoleucine	(g)	0.8184	0.7645
Leucine	(g)	1.3995	1.5692
Lysine	(g)	1.2685	1.3601
Methionine	(g)	0.3601	0.2915
Cyteine	(g)	0.1105	0.4541
Phenylalanine	(g)	0.7366	0.5681
Tyrosine	(g)	0.6138	0.5111
Threonine	(g)	0.622	0.9567
Tryptophan	(g)	0.1923	0.2598
Valine	(g)	0.9821	0.8554
Arginine	(g)	0.491	1.3580
Histidine	(g)	0.4051	0.3878
Alanine	(g)	0.491	0.7202
Aspartic acid	(g)	1.1867	1.5016
Glutamic acid	(g)	2.8644	2.3485
Glycine	(g)	0.2946	0.3612
Proline	(g)	1.3913	0.8195
Serine	(g)	0.8184	0.9757
Sum	(g)	15.05	16.06

Supplementary Table S2. The number of subjects included in data analysis for selected characteristics by treatment group. Fifty samples per each group were initially chosen by stratified random sampling, matched for age, the number of anemic subjects, and the number of females or males in each group. The samples with excessive dilution, or noisy spectra were excluded from the analysis, leaving 46 (MFGM group) and 45 (Control group) subjects respectively.

	**Total number of samples included in analysis**	**Age at entry** *	**Anemic**	**Sex (F / M)**
**MFGM**	46	8.2 ± 1.8	24	18 / 28
**Control**	45	8.5 ± 1.7	21	21 / 24

*Average ± S.D.

Supplementary Table S3. The concentration of several metabolites differed between the MFGM and the control group in all, male (n = 28 (MFGM), 24 (control)) and female infants (n = 18 (MFGM), 21 (control)). Concentrations are reported in µM as median and interquartile range (3rd - 1st). IQR = interquartile range.

	All Infants				Male Infants				Female Infants			
	MFGM		Control		MFGM		Control		MFGM		Control	
**Metabolite**	**Median**	**IQR**	**Median**	**IQR**	**Median**	**IQR**	**Median**	**IQR**	**Median**	**IQR**	**Median**	**IQR**
Essential amino acids												
Isoleucine	86.7	35.4	78.3	34	87.1	36.4	84.9	43.3	84.3	27.8	73.7	23.6
Leucine	136.4	67	119.3	56	136.3	53.1	129.2	55.7	144.6	63.1	106.8	41.9
Lysine	179.6	55.5	164.3	37.8	177.6	70.7	162.6	55.2	193.0	36.8	164.3	28.9
Phenylalanine	57.1	21.5	58.2	18.8	57.1	14.1	61.1	22.9	62.2	25.6	56.2	11.3
Threonine	135.2	54.5	123.8	38.6	118.8	55.4	126.1	43.6	145.2	25.0	115.1	32.2
Valine	250	94.9	216	76.3	247.9	100.7	221.3	72.4	251.8	72.7	216.0	71.4
Non-essential amino acids												
Alanine	448.1	93.3	424.1	167.5	456.7	113	458.9	150.8	439.4	149.8	374.4	137.9
Arginine	179.3	57.6	158.7	58.1	166.6	44.1	157.8	53.9	201.6	49.4	166.1	64.7
Asparagine	13.3	7.7	15.7	9.6	15.6	7.7	17.0	9.3	13.1	6.9	13.9	6.6
Proline	277.6	98	257.7	93.6	257.2	101.7	264.7	82.0	279.0	1060	236.9	103.3
Serine	206.6	46.2	206	33.4	201.8	46.4	204.5	40.4	212.6	46.7	211.4	29.9
Tyrosine	93.3	32.4	85.9	27.3	93.2	33.3	85.6	46.8	98.3	26.8	88.3	24.5
Urea cycle intermediates												
Ornithine	66.1	23.2	60.6	21.4	61.2	25.4	67.6	23.7	72.7	10.1	59.6	13.6
Urea	2003.4	936.4	1423.9	676.7	1999.6	1010.8	1513.9	702.7	2007.2	844.1	1402.0	583.9
Ketone body metabolites												
Acetone	26.9	16.6	30.7	51.7	27.1	17.1	28.3	14.9	24.7	21.7	65.6	85.8
3-OHB	60.4	80.7	82.6	199.4	49.4	80.1	60.9	66.0	72.9	109.9	215.1	302.8
Other metabolites												
Aspartate	75.5	24.9	73.7	19.2	73.1	19.4	73.6	19.5	86.0	37.1	75.3	19.2
Creatine	75	24.4	70.5	21	83.0	24.6	73.0	16.5	70.2	12.8	65.3	19.7
Choline	36	15	29.7	13.3	35.0	10.4	30.8	12.1	44.9	14.6	26.6	16.7
DMA	4.1	1.1	3.8	1.2	4.2	0.8	3.9	1.3	3.9	1.1	3.8	0.9
Mannose	35.6	12.1	30.8	10.1	36.1	13.0	30.9	8.9	32.9	10.3	29.9	13.8
N,N-DMG	4.7	3.9	5.2	5	4.4	2.8	6.1	4.7	5.1	4.1	4.6	3.9
PG	5.4	16.3	8.4	21.1	5.8	25.4	10.1	33.1	3.4	4.2	8.3	10.9
Pyroglutamate	137.6	59.3	117.4	52.6	125.9	40.2	117.4	38.6	175.6	56.0	115.8	62.8
Succinate	10.2	3.9	9.6	2.6	10.1	4.0	9.0	2.9	10.8	4.1	9.7	1.9
Taurine	129.5	57.1	108.8	59.3	126.6	54.3	109.9	65.8	151.6	60.9	103.6	77.1
TMAO	4.3	2.4	7.8	2.9	4.2	2.2	7.5	2.2	4.8	2.6	8.2	2.7

Abbreviations: 3-Hydroxybutyrate (3-OHB), Dimethylamine (DMA), N,N-Dimethylglycine (N,N-DMG), Propylene glycol (PG), Trimethylamine-N-oxide (TMAO).

Supplementary Table S4. The metabolites with identified sex effect were analyzed by a linear regression model with sex and diet as fixed effect factors, and significance of diet (MFGM supplementation) adjusted for sex was computed. Additional statistics from the linear model as well as the slope coefficient estimates for Sex.Male effect (the magnitude of metabolite-level changes when the fixed class variable changes from female to male) and Diet.MFGM effect are summarized.

Metabolite	Adjusted R^2	F-statistics	Intercept	p-value for Sex	p-value for Sex*Diet	Estimated slope coeff. for Sex.male	Estimated slope coeff. for Diet.MFGM
Essential amino acids							
Isoleucine	-0.002	0.952	1.868	0.252	0.295	0.051	0.074
Leucine	0.018	1.541	2.064	0.370	0.195	0.039	0.098
Lysine	0.020	1.603	2.210	0.746	0.186	0.011	0.075
Phenylalanine	0.007	1.205	1.732	0.303	0.230	0.036	0.070
Threonine	-0.006	0.839	2.077	0.742	0.418	0.013	0.062
Valine	0.011	1.313	2.319	0.232	0.223	0.045	0.076
Non-essential amino acids							
Alanine	0.011	1.313	2.319	0.232	0.223	0.045	0.076
Arginine	0.062	2.900	2.236	0.404	0.337	-0.027	0.070
Asparagine	0.008	1.241	1.146	0.173	0.330	0.094	-0.013
Proline	0.018	1.528	2.385	0.202	0.070#	0.047	0.084
Serine	-0.018	0.479	2.333	0.527	0.779	-0.015	0.006
Tyrosine	0.016	1.462	1.926	0.067#	0.084#	0.067	0.069
Urea cycle intermediates							
Ornithine	0.023	1.688	1.773	0.222	0.045*	0.045	0.086
Urea	0.047	2.442	3.136	0.598	0.873	0.031	0.115
Ketone body metabolites							
Acetone	0.071	3.214	1.692	0.038*	0.200	-0.219	-0.268
3-OHB	0.092	3.931	2.194	0.013*	0.218	-0.366	-0.356
Other metabolites							
Aspartate	0.022	1.662	1.862	0.813	0.099#	0.008	0.067
Creatine	0.051	2.561	1.819	0.356	0.548	0.029	0.025
Choline	0.036	2.084	1.526	0.487	0.605	-0.039	0.104
DMA	-0.013	0.633	0.527	0.807	0.891	-0.014	0.048
Mannose	0.042	2.274	1.470	0.686	0.924	0.014	0.064
N,N-DMG	-0.031	0.130	0.696	0.629	0.572	0.039	0.245
PG	0.029	1.877	0.934	0.423	0.515	0.124	-0.253
Pyroglutamate	0.019	1.565	2.083	0.991	0.194	-0.001	0.097
Succinate	0.020	1.593	0.986	0.391	0.118	-0.030	-0.010
Taurine	0.004	1.113	1.991	0.210	0.106	0.071	0.108
TMAO	0.317	14.470	0.917	0.286	0.562	-0.062	-0.288

Group differences with approaching significance (# = $0.05 < P < 0.1$) or significance (* = $0.1 < P < 0.05$) were noted.

The residual plots (as diagnostic plots) generated from the linear regression model were reported in the following data repository:

Click on the link
or go to:
https://drive.google.com/drive/folders/0B3cVdIJxMYUOei1STHVrZUF0QkE?usp=sharing

Supplementary Table S5. Correlation coefficient (ρ) and *P*-value from Spearman correlation analysis of the selected serum metabolites, cytokines, and micronutrient status. *P* values in between 0.05 > *P* > 0.01 are marked in yellow, and less than *P* < 0.01 marked in red. Correlation coefficient (ρ) > 0.8 marked in dark blue, 0.6 < ρ < 0.8 in sky blue, and 0.4 < ρ < 0.6 in pale blue.

P-value \ Coeff.(ρ)	Leucine	Isoleucine	Lysine	Valine	Phenylalanine	Isobutyrate	Isovalerate	Tyrosine	2-Hydroxybutyrate	3-Hydroxyisobutyrate	Arginine	Urea	Creatine	Acetone	3-Hydroxybutyrate	Lactate	TMAO	Propylene glycol	IL-2	IL-6	IFN-g	IL-5	IL-10	Zinc	Hb	folate	ferrtin	B12	Age
Leucine		0.95	0.92	0.90	0.81	0.76	0.83	0.84	0.61	0.65	0.56	0.51	0.70	0.25	0.09	0.17	-0.12	0.03	-0.34	-0.28	-0.06	0.03	-0.13	-0.05	0.00	-0.01	0.07	0.16	0.10
Isoleucine	7.7E-21		0.89	0.89	0.75	0.71	0.84	0.79	0.57	0.62	0.48	0.49	0.71	0.25	0.08	0.12	-0.15	0.13	-0.26	-0.19	-0.02	0.03	-0.10	-0.10	-0.05	0.02	-0.05	0.13	0.00
Lysine	3.7E-17	0.00		0.80	0.71	0.76	0.83	0.80	0.58	0.62	0.69	0.46	0.61	0.30	0.16	0.01	-0.02	0.04	-0.29	-0.27	0.02	0.06	-0.04	-0.19	-0.11	0.10	0.12	0.14	-0.02
Valine	2.3E-15	1.4E-14	3.9E-10		0.76	0.74	0.69	0.89	0.68	0.73	0.42	0.73	0.77	0.05	-0.11	0.19	-0.15	-0.16	-0.36	-0.20	-0.03	0.05	-0.18	-0.12	0.12	-0.04	0.01	0.18	0.17
Phenylalanine	3.0E-10	1.4E-14	3.5E-07	1.7E-08		0.65	0.60	0.64	0.52	0.53	0.49	0.55	0.68	0.04	-0.13	0.23	-0.11	-0.14	-0.43	-0.23	-0.13	-0.13	-0.17	0.02	-0.03	-0.04	0.13	0.23	0.23
Isobutyrate	1.6E-08	3.3E-07	1.3E-08	6.4E-08	5.9E-06		0.84	0.74	0.78	0.82	0.35	0.57	0.61	0.32	0.25	0.06	0.06	-0.17	-0.45	-0.46	-0.11	-0.12	-0.21	-0.01	-0.02	0.29	0.18	0.13	0.08
Isovalerate	2.5E-11	2.0E-11	5.4E-11	6.4E-08	4.4E-05	1.2E-11		0.72	0.63	0.68	0.37	0.31	0.50	0.52	0.45	-0.02	0.02	0.13	-0.31	-0.41	-0.13	-0.11	-0.19	-0.08	-0.15	0.18	0.15	0.06	-0.02
Tyrosine	9.5E-12	6.2E-08	3.9E-08	2.9E-14	9.6E-06	6.1E-08	1.3E-07		0.62	0.79	0.46	0.63	0.60	0.18	0.06	0.03	-0.10	-0.09	-0.39	-0.23	0.07	0.06	-0.16	-0.13	0.02	-0.01	0.15	0.14	0.06
2-Hydroxybutyrate	3.1E-05	6.2E-08	3.3E-03	1.2E-06	5.9E-04	2.6E-09	1.3E-05	2.2E-05		0.80	0.20	0.71	0.61	0.18	0.11	0.00	0.01	-0.31	-0.38	-0.40	-0.13	-0.10	-0.36	-0.03	0.17	0.32	0.22	0.21	0.26
3-Hydroxyisobutyrate	6.3E-06	2.0E-05	2.1E-05	8.7E-08	4.0E-04	1.5E-10	1.5E-06	1.1E-09	7.9E-10		0.14	0.74	0.61	0.21	0.19	0.00	0.02	-0.23	-0.48	-0.39	-0.17	-0.16	-0.26	-0.06	0.06	0.26	0.14	0.04	0.13
Arginine	1.9E-04	2.1E-03	2.1E-06	6.9E-03	1.4E-03	0.03	0.02	2.8E-03	0.22	0.38		0.21	0.25	0.03	-0.04	-0.06	-0.08	-0.07	-0.08	-0.01	0.27	0.25	0.25	-0.06	-0.15	0.05	0.23	0.06	-0.03
Urea	8.8E-04	1.5E-03	3.3E-03	9.5E-08	2.7E-04	1.4E-04	0.0494	2.2E-05	2.2E-07	5.7E-08	0.20		0.69	-0.20	-0.32	0.13	-0.19	-0.49	-0.36	-0.18	-0.03	0.13	-0.19	0.01	0.11	0.17	0.03	0.32	0.37
Creatine	4.8E-07	7.5E-07	3.7E-05	6.7E-09	1.5E-06	2.7E-05	1.1E-03	5.7E-05	3.4E-05	3.0E-05	0.12	2.E-06		-0.04	-0.27	0.38	-0.03	-0.06	-0.31	-0.11	-0.04	-0.04	-0.07	-0.06	0.17	-0.07	-0.08	0.13	0.27
Acetone	0.12	0.13	0.06	0.77	0.83	0.04	5.2E-04	0.27	0.27	0.19	0.85	0.22	0.81		0.85	-0.23	0.29	0.30	-0.04	-0.23	-0.16	-0.13	-0.07	0.01	-0.09	0.13	0.03	-0.33	-0.13
3-Hydroxybutyrate	0.59	0.62	0.34	0.48	0.43	0.12	3.5E-03	0.74	0.49	0.24	0.80	0.05	0.09	4.E-12		-0.40	0.33	0.23	-0.05	-0.35	-0.23	-0.17	-0.04	0.02	-0.22	0.22	0.13	-0.31	-0.16
Lactate	0.31	0.45	0.94	0.24	0.15	0.69	0.90	0.86	0.98	0.98	0.73	0.42	0.02	0.15	0.01		-0.24	0.11	-0.14	0.13	0.13	0.09	0.07	-0.01	0.30	-0.34	-0.31	0.11	0.03
TMAO	0.45	0.35	0.88	0.35	0.48	0.71	0.91	0.54	0.93	0.89	0.64	0.23	0.85	0.07	0.04	0.14		0.00	-0.16	-0.41	-0.25	-0.33	-0.23	0.12	-0.13	-0.09	0.10	-0.42	0.03
Propylene glycol	0.86	0.43	0.81	0.33	0.39	0.30	0.44	0.58	0.05	0.16	0.67	1.2E-03	0.71	0.06	0.16	0.50	0.98		0.41	0.29	0.22	-0.07	0.26	-0.15	-0.19	0.02	-0.17	-0.06	-0.44
IL-2	0.03	0.11	0.07	0.02	0.01	3.4E-03	0.05	0.01	0.02	1.6E-03	0.64	0.02	0.05	0.79	0.75	0.39	0.32	0.01		0.63	0.21	0.36	0.43	0.01	0.05	0.01	-0.13	0.06	-0.33
IL-6	0.08	0.25	0.09	0.21	0.16	3.0E-03	0.01	0.15	0.01	0.01	0.95	0.26	0.50	0.15	0.03	0.44	0.01	0.07	1.E-05		0.40	0.30	0.56	-0.15	0.03	-0.17	-0.26	-0.04	-0.27
IFN-g	0.73	0.90	0.90	0.86	0.44	0.49	0.42	0.66	0.41	0.31	0.10	0.86	0.80	0.33	0.16	0.43	0.12	0.17	0.19	0.01		0.26	0.55	-0.30	-0.11	-0.01	0.01	0.34	-0.09
IL-5	0.83	0.83	0.73	0.76	0.43	0.46	0.49	0.71	0.53	0.32	0.12	0.43	0.83	0.44	0.30	0.56	0.04	0.66	0.02	0.06	0.11		0.33	0.15	-0.12	-0.05	-0.06	0.40	0.05
IL-10	0.42	0.54	0.82	0.27	0.30	0.18	0.24	0.31	0.02	0.10	0.11	0.24	0.67	0.66	0.81	0.65	0.15	0.11	0.01	1.9E-04	3.3E-04	0.03		-0.16	-0.15	0.00	-0.21	-0.03	-0.05
Zinc	0.74	0.56	0.25	0.46	0.89	0.94	0.64	0.42	0.86	0.72	0.71	0.96	0.73	0.96	0.91	0.93	0.45	0.35	0.97	0.35	0.06	0.34	0.32		0.02	0.04	0.06	0.01	0.19
Hb	0.98	0.75	0.48	0.45	0.84	0.89	0.37	0.91	0.30	0.69	0.38	0.48	0.30	0.56	0.18	0.06	0.43	0.24	0.76	0.85	0.49	0.47	0.35	0.89		-0.19	-0.15	-0.17	0.12
folate	0.96	0.91	0.54	0.81	0.81	0.07	0.26	0.95	0.04	0.69	0.74	0.29	0.68	0.44	0.16	0.03	0.59	0.91	0.95	0.31	0.96	0.78	1.00	0.80	0.25		0.00	0.12	0.11
ferrtin	0.49	0.94	0.54	0.85	0.15	0.26	0.18	0.42	0.09	0.25	0.67	0.78	0.72	0.57	0.27	0.10	0.48	0.55	0.49	0.04	0.49	0.15	0.02	0.34	0.49	0.91		0.27	0.00
B12	0.33	0.42	0.39	0.28	0.15	0.43	0.71	0.39	0.19	0.80	0.71	0.04	0.42	0.04	0.05	0.48	0.01	0.70	0.70	0.79	0.03	0.01	0.85	0.95	0.29	0.45	0.05		0.23
Age	0.56	0.99	0.89	0.29	0.16	0.62	0.89	0.72	0.11	0.41	0.85	0.02	0.09	0.42	0.31	0.86	0.85	4.9E-03	0.04	0.09	0.57	0.77	0.77	0.25	0.47	0.52	0.83	0.16	

A **Metabolite Set Enrichment Analysis overview – serum**

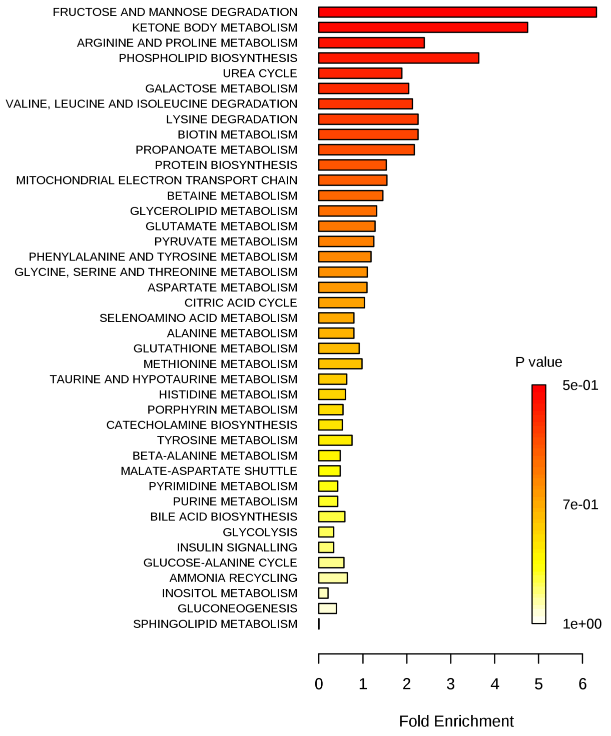

B

Rank	Metabolite set	Associated metabolites	Raw p
1	Fructose and mannose de gradation	***Mannose***	1.12E-02
2	Ketone body metabolism	3- Hydroxybutyric acid, Acetone	2.85E-02
3	Arginine and proline metabolism	***Arginine, urea, proline, creatine, creatinine, ornithine,*** glutamine	3.80E-02
4	Phospholipid biosynthesis	***Choline***	5.60E-02
5	Urea cycle	***Arginine, urea, ornithine***, glutamine	8.51E-02
6	Galactose metabolism	***Glycerol, mannose***	8.98E-02
7	Valine, leucine and isoleucine degradation	***Valine, leucine, 3-hydroxyisobutyric acid***	1.30E-01
8	Lysine degradation	***Lysine***	1.34E-01
9	Biotin metabolism	***Lysine***	1.34E-01
10	Propanoate metabolism	***Valine***	1.41E-01
11	Protein biosynthesis	***Proline, arginine, valine, leucine***, tyrosine, ***phenylalanine,***	1.66E-01
12	Mitochondrial electron transport chain	Fumaric acid, ***succinic acid***	2.12E-01
13	Betaine metabolism	***Choline,*** methionine, dimethylglycine, betaine	2.23E-01
14	Glycerolipid metabolism	***Glycerol***	2.54E-01

*The metabolites higher in the MFGM group are highlighted in **bold and *Italic***

Supplementary Figure S6. (A) Summary of Metabolite Set Enrichment Analysis (MSEA). *P* values are indicated as color (red: lowest *P* value) and fold enrichment as a length of each bar. (B) Associated metabolites that are enriched in a corresponding metabolite set. The metabolites higher in the MFGM group are highlighted in **bold** and *Italic*.

A **Metabolic Pathway Enrichment Analysis (MPEA) summary**

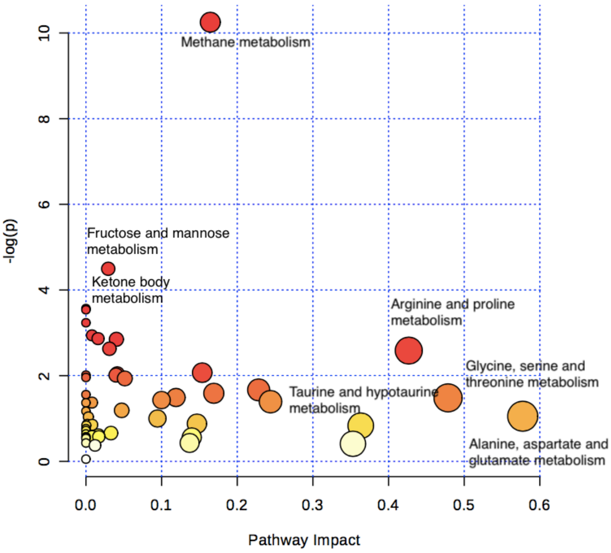

B

KEGG pathway	Associated metabolites
Methane metabolism	Trimethylamine-N-oxide, ***dimethylamine***, formic acid
Fructose and mannose metabolism	Mannose
Pyrimidine metabolism	***Urea***, glutamine
Synthesis and degradation of ketone bodies	***3- Hydroxybutyric acid***, acetone
Amino sugar and nucleotide sugar metabolism	Glucose, mannose
Purine metabolism	Glycine, ***urea***, glutamine
Propanoate metabolism	***Valine***, acetone, 2-hydroxybutyric acid, succinic acid, lactic acid
Butanoate metabolism	Succinic acid, fumaric acid, 3- hydroxybutyric acid
Glycerophospholipid metabolism	Glycerophosphocholine, choline
Arginine and proline metabolism	***Arginine, urea, proline,*** creatine, creatinine, ***ornithine***, glutamine
Glyoxylate and dicarboxylate metabolism	Glycolic acid, formic acid, succinic acid, citric acid
Valine, leucine and isoleucine degradation	***Valine, leucine***, 3-hydroxyisobutyrate
Valine, leucine and isoleucine biosynthesis	***Valine, leucine, isoleucine,*** threonine
Biotin metabolism	***Lysine***
D-Arginine and D-ornithine metabolism	***Arginine, ornithine*** (L-form)

*The metabolites higher in the MFGM group are highlighted in **bold and *Italic***

Supplementary Figure S7. (A) Metabolic pathways significantly enriched by differences in the metabolome between the control and MFGM group. Enrichment scores are represented according to their log(p) values (y-axis) and pathway impacts (x-axis). (B) The associated metabolites that ”hit” the corresponding metabolites in a KEGG library pathway are listed.
